# ﻿The first report of two new janiroid isopod species (Asellota, Janiroidea) from the western Indian Ocean

**DOI:** 10.3897/zookeys.1219.130895

**Published:** 2024-12-03

**Authors:** Valiallah Khalaji-Pirbalouty, Manal Abdulrahman Al-Kandari

**Affiliations:** 1 Department of Biology, Faculty of Basic Science, Shahrekord University, Shahrekord, Iran Shahrekord University Shahrekord Iran; 2 Coastal and Marine Research Program, Environment and Life Sciences Research Center, Kuwait Institute of Scientific Research, Hamad Al-Mubarak Street, Building 900004, Area 1, Raas Salmiya, Kuwait Environment and Life Sciences Research Center, Kuwait Institute of Scientific Research Raas Salmiya Kuwait

**Keywords:** *Heterosignum*, identification key, Indian Ocean, Kuwait, Munnidae, new record, new species, Paramunnidae, taxonomy, *Uromunna*

## Abstract

Two new janiroid isopod species, *Heterosignumbehbehanii***sp. nov.** and *Uromunnaalyamanii***sp. nov.**, are described from the Kuwaiti waters, representing the first record of the genera in the western Indian Ocean. *Heterosignumbehbehanii***sp. nov.** is distinguished from its congeners by pereonites 2–7 with a pair of short single lateral spine-like processes; the relatively short length of the anterior part of its pleotelson, which accounts for about 0.1 of the total length of the pleotelson; and the stylet with an elongate, downwardly curved, and distally pointed apex. *Uromunnaalyamanii***sp. nov.** is equally unique, with the pleotelson bearing two denticles on each lateral margin, pleopod 2 endopod stylet extending beyond the apex of the protopod, and pleopod 4 exopod distal tip with a long plumose seta. This discovery of unique characteristics of the new species significantly enhances our understanding of marine biodiversity in the western Indian Ocean and underscores the importance of further research in this area. The identification keys are provided to all known species of *Heterosignum* and Indian Ocean *Uromunna* species.

## ﻿Introduction

The superfamily Janiroidea Sars, 1897 is composed of 25 families, with Paramunnidae Vanhöffen, 1914 and Munnidae Sars, 1897, standing out as the two largest and most diverse families within the suborder Asellota (Boyko et al. 2008, onwards). The isopod family Paramunnidae consists of about 195 described species classified into 45 genera, all of which are tiny benthic dwellers known worldwide, from shallow tidal zones to abyssal depths (Just and Wilson 2007; Boyko et al. 2008, onwards). Within Paramunnidae, the genus *Heterosignum* Gamô, 1976, comprises seven valid species. Apart from *Heterosignumunicornis* (Kensley, 1976), found in the Amsterdam Islands in the southern Indian Ocean, the remaining *Heterosignum* species have been exclusively reported in the areas around Japan (Shimomura 2009, 2011).

The family Munnidae Sars, 1897, with its 114 described species distributed across six genera (Boyko et al. 2008, onwards), is a testament to the adaptability of these small, free-living isopods. They thrive in diverse environments, ranging from shallow waters to abyssal depths spanning tropical and polar regions across the globe. Their remarkable adaptability allows them to thrive in various habitats, including mud and sand flats, intertidal rock pools, seagrass meadows, sponges, and coral reefs (Wilson 1980). This adaptability is a fascinating aspect of their biology that continues to intrigue researchers in the field. Within Munnidae, the genus *Munna* Krøyer, 1839, is the most species-rich and is currently composed of 79 valid species (Boyko et al. 2008 onwards). The genus *Uromunna* Menzies, 1962, is the second known genus with the highest species diversity of 28 recorded species (Boyko et al. 2008, onwards).

To date, 15 *Uromunna* species have been reported from the Atlantic Ocean, primarily in the northern regions, and 10 species in the Pacific Ocean. Notably, the eastern Indian Ocean, particularly the Australian region, is home to three *Uromunna* species: *U.brevicornis* (Thomson, 1946) from Swan River, Western Australia; *U.humei* Poore, 1984 from Apollo Bay, Victoria, and *U.phillipi* Poore, 1984 from Port Phillip Bay, Victoria. However, no *Uromunna* species have been reported from the western, northern, and southern regions of the Indian Ocean. This absence makes our discovery of two new species, one each from *Heterosignum* and *Uromunna* in the western Indian Ocean, particularly significant.

## ﻿Materials and methods

Sample collection was conducted during sampling trips (2013–2017), focusing on the littoral zone along Kuwait’s coastline and offshore islands (Al-Kandari et al. 2022). All material was fixed and preserved in 96% ethanol.

The specimens’ sorting, dissection and imaging were performed using a Leica® M125 stereomicroscope equipped with a DFC450 camera. Dissected appendages were mounted onto glass slides in stained antibacterial glycerine-gelatine (Merck). Pencil appendage drawings were made using a Leica (DM1000) compound microscope equipped with camera lucida. All illustrations were electronically inked with Corel Draw (version X6). The specimens were prepared for SEM photographs using previously described techniques (Khalaji-Pirbalouty et al. 2022) and examined with a Vega3 SBU scanning electron microscope (TESCAN Brno, Czech Republic).

The morphological terminology used herein is a comprehensive compilation from established sources such as Poore (1984), Shimomura and Mawatari (2002), and Esquete and Wilson (2016). All the material used in this study is deposited in the Museum of Nature, Hamburg, the Leibniz Institute for the Analysis of Biodiversity Change (**LIB **).

## ﻿Results

### ﻿Taxonomy

**Suborder Asellota Latreille, 1802**

**Superfamily Janiroidea Sars, 1897**

**Family Paramunnidae Vanhöffen, 1914**

#### ﻿Genus Heterosignum

Taxon classificationAnimaliaIsopodaParamunnidae

Gamô, 1976

9216169D-0D70-560D-8824-544D0DD3C5A7

##### Type species.

*Heterosignummutsuensis* Gamô, 1976 (original designation).

##### Diagnosis.

The most recent revision and diagnosis of the genus is that of Shimomura and Mawatari (2002).

#### ﻿ Heterosignum behbehanii sp. nov.

Taxon classificationAnimaliaIsopodaParamunnidae

FF97B31C-FD94-56E7-BB85-76D95DF7F205

https://zoobank.org/38B4B177-F22A-42D0-A139-61D9D21B8F38

**Figure 1.**
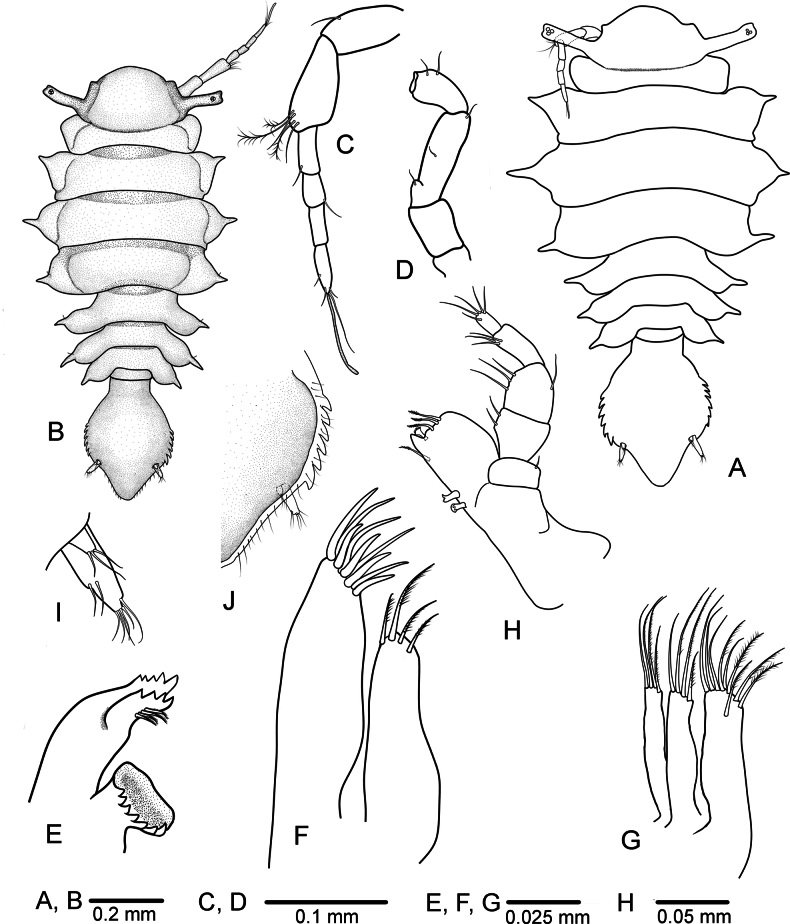
*Heterosignumbehbehanii* sp. nov. **A** female habitus **B–J** male holotype (ZMH-K-64934) **B** habitus **C** antennula **D** antenna **E** mandible **F** maxillula **G** maxilla **H** maxilliped **I** uropod **J** pleotelson lateral margin, ventral view.

**Figure 2.**
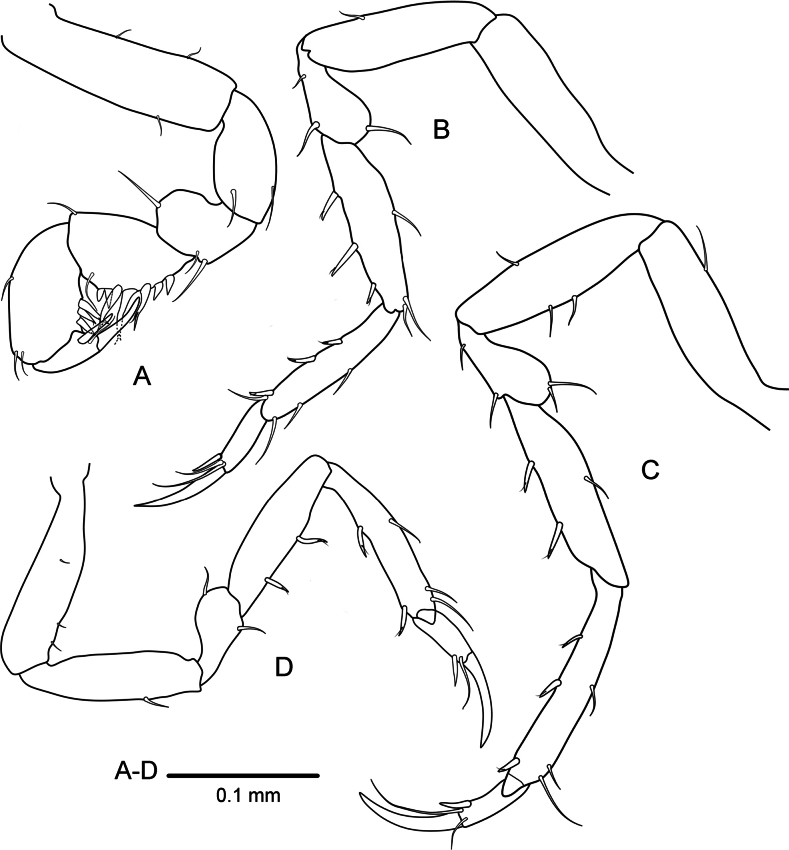
*Heterosignumbehbehanii* sp. nov. (ZMH-K-64934), male holotype **A** pereopod 1 **B** pereopod 2 **C** pereopod 5 **D** pereopod 7.

**Figure 3.**
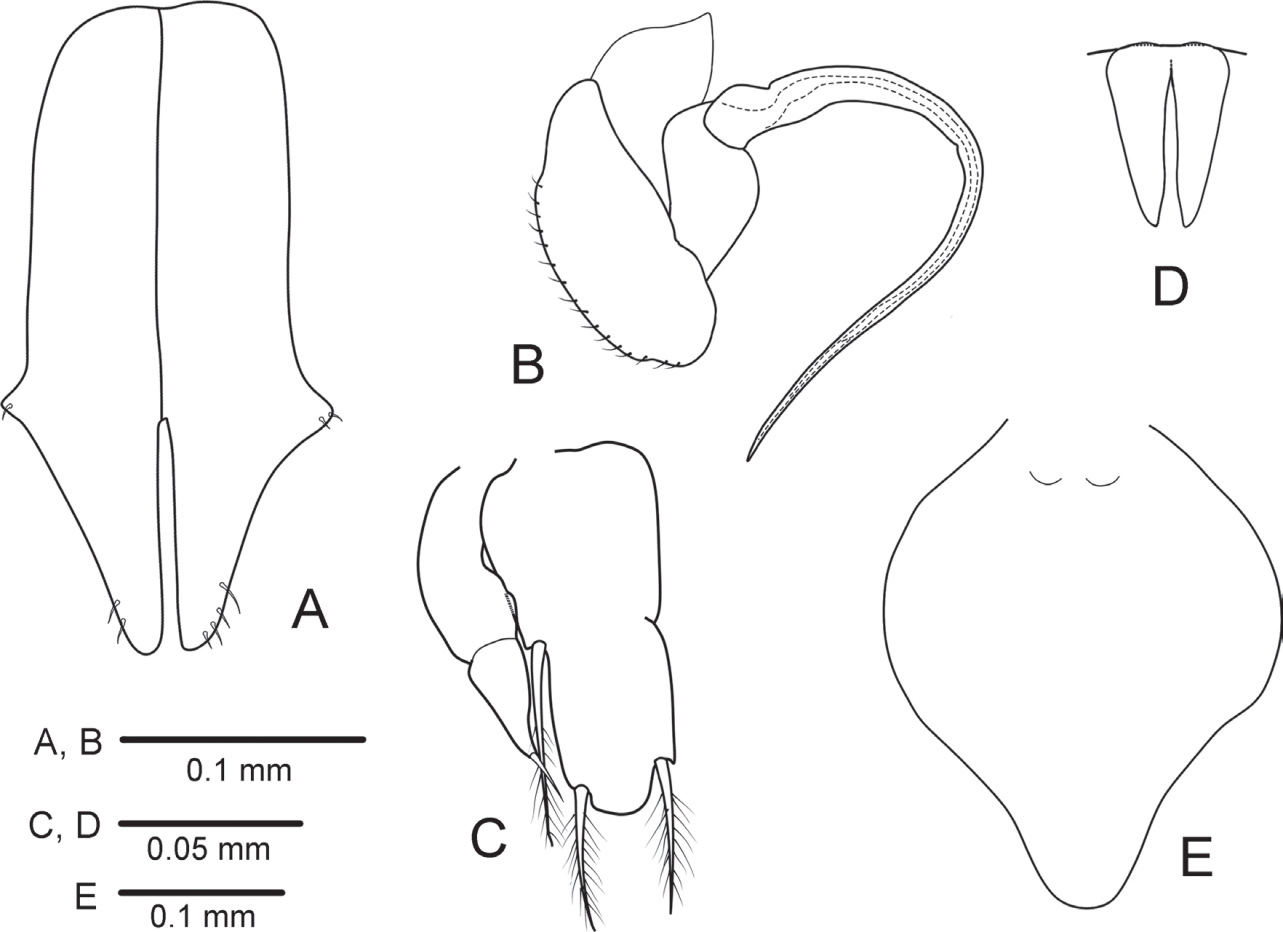
*Heterosignumbehbehanii* sp. nov. (ZMH-K-64934), male holotype **A** pleopods 1 **B** pleopod 2 **C** pleopod 3 **D** penial processes **E** female operculum.

**Figure 4.**
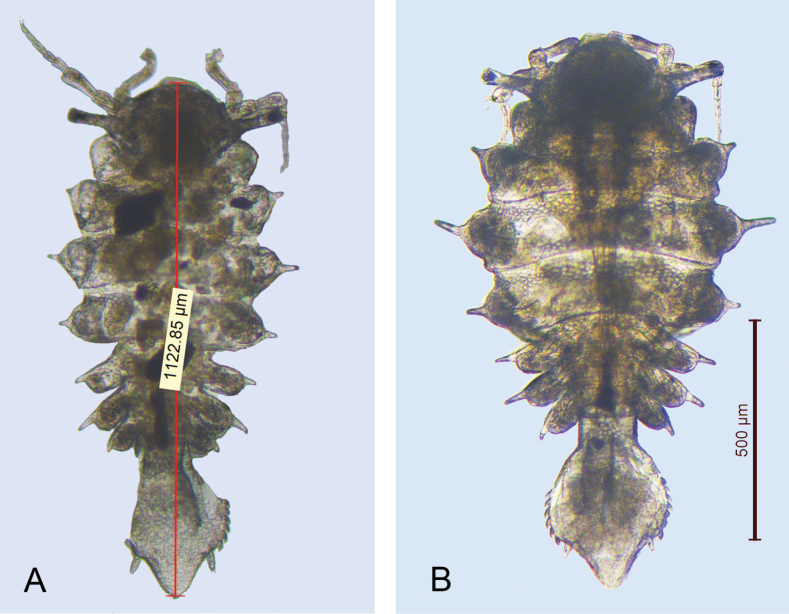
*Heterosignumbehbehanii* sp. nov. Al-Khiran, Kuwait (28°38.484'N, 48°23.287'E) **A** male **B** female.

**Figure 5.**
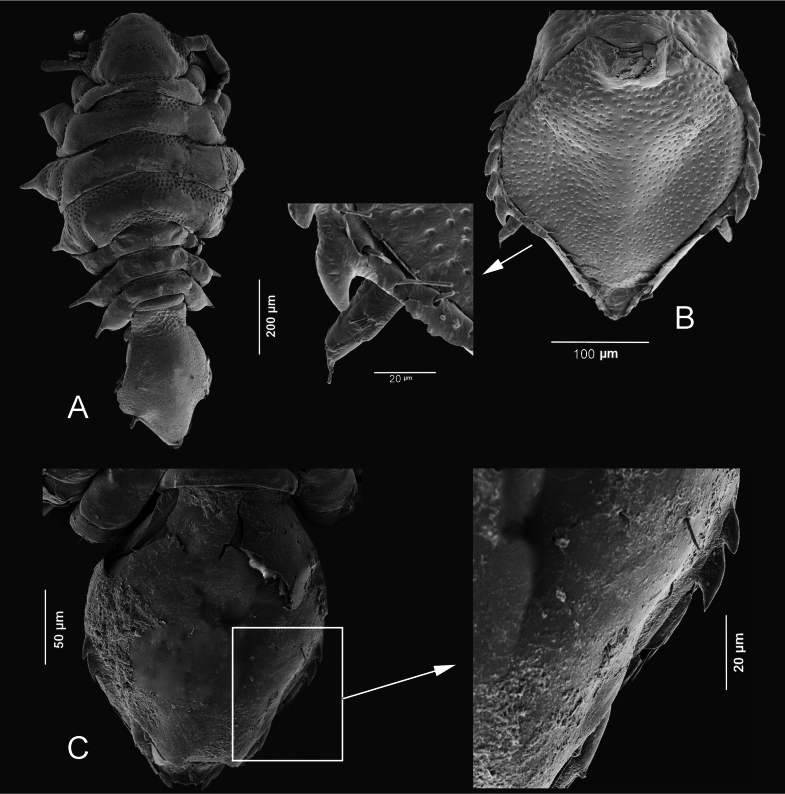
Scanning electron micrographs **A, B***Heterosignumbehbehanii* sp. nov. (Al-Khiran, Kuwait: 28°38.484'N, 48°23.287'E) **A** female habitus **B** pleotelson, ventral view **C***Uromunnaalyamanii* sp. nov. (Al-Nuwaseeb, Kuwait: 28°34.792'N, 48°24.078'E), female pleotelson.

Figs 1 , 2 , 3 , 4 , 5A, B

##### Etymology.

The species is named in honour of Dr Abdulmanaf Behbehani, a respected figure who taught Marine Biology and Ecology at Kuwait University and researched Kuwait's marine environment and Kuwait's intertidal macrofauna for over four decades.

##### Type material.

***Holotype***: • ♂, 1.12 mm (ZMH-K-64934), KUWAIT, Al-Salam Beach; 29°21.631'N, 47°57.204'E; 28.X.2014; V. Grintsov leg. ***Paratypes***: • 1♀, 1.34 mm (ZMH-K-64935), same data as the holotype • 1 ♂ 1.1 mm; 4 ♀♀ up to 1.25 mm (ZMH-K-64936), Umm Al-Maradim Island; 28°40.778'N, 48°39.207'E; 11.XI.2014; V. Grintsov leg.

##### Diagnosis.

Eyes with 3 ommatidia; eyestalks with medium length, reaching proximal half of peduncular article 2 of antenna 2. Pereonites 2–7 with a pair of relatively short single lateral spine-like processes. The pleotelson has a relatively short anterior neck, length about 0.1 pleotelson length, lateral margins with 6 denticles each. Male pleopod 2 with an elongate, downwardly curved and distally pointed apex endopodal stylet.

##### Description.

**Male**: Body (Fig. 1B) length 1.28 mm; about 2 times as long as wide, widest at pereonite 3.

***Head*** (Fig. 1B) 0.36 times as long as wide (including eyestalks); frontal margin convex, having pair of weak preocular lobes. ***Eyestalks*** (Figs 1B, 4A) arising from posterior half of head, reaching proximal half of peduncular article 2 of antenna 2, 0.62 length of head, eyes with 3 ommatidia.

***Pereon*** (Figs 1B, 4A, 5A) dorsal surface smooth; ***pereonite 1*** laterally rounded, without lateral spine-like processes; ***pereonites*** 2–7 with a pair of short single lateral spine-like processes; ***pereonites*** 1–3 increasing in width and ***pereonites*** 4–7 decreasing in width. ***Pleonite 1*** small, 0.26 times as long as wide, with pair of fine dorsal setae.

***Pleotelson*** (Fig. 1B, J) about 1.3 times longer than wide, anterior part narrow, about 0.13 times of total length of pleotelson, posterior part bulbous, tapering posteriorly, lateral margins each with 6 denticles, posterior margin rounded, ventral margins with a raw of small submarginal simple setae. ***Uropod*** (Fig. 1I) cylindrical, endopod with 2 lateral and 6 apical setae; exopod about 0.3 length of endopod, with 3 apical setae.

***Antennula*** (Fig. 1C) peduncular article 1 shorter than article 2; article 2 with 1 simple and 3 small sensory palmate setae distodorsally; article 3 about 0.5 length of article 2, with 1 simple seta distally; flagellum with three articles, article 1 shortest, with 1 distoventral seta; article 3 subequal to article 2, distally with 1 long, 2 short setae and 1 aesthetasc.

***Antenna*** (Fig. 1D) with peduncle articles 1 and 2 combined about 0.8 length of article 3; peduncle article 3 with convex proximal protrusion bearing single seta, with 1 simple seta medially and 1 simple seta distally; article 4 about half length of article 3, with 2 distolateral setae.

***Mandible*** (Fig. 1E) incisor process with 5 and ***lacinia mobilis*** with 4 cusps; spine row with 4 robust serrate setae.

***Maxillula*** (Fig. 1F): lateral lobe with 9 robust apical setae; medial lobe with 4 robust serrate setae on distal margin.

***Maxilla*** (Fig. 1G) lateral and mesial lobes, each with 4 pectinate robust apical setae; medial lobe with 9 robust setae along distal margin, some of them serrated.

***Maxilliped*** (Fig. 1H) endite distal margin with 1–2 fan-shaped setae, 3 serrate setae, and 1 sub marginal seta medially, mesial margin with 2 coupling hooks; palp article 1 with 1 short seta laterally; article 2 shorter than article 3 with 2 setae medially; article 3 with 3 setae medially and 1 seta laterally; article 4 subequal in length to article 3, with 3 setae distomedially; article 5 narrowest, with 5 setae distally.

***Pereopod 1*** (Fig. 2A) shorter than ***pereopods 2*–*7***: basis 3.6 times as long as greatest width, superior margin with 1 and inferior margin with 2 small setae; ischium 0.5 times as long as basis, with 2 long setae distally; merus shorter than carpus, superior margin with 1 robust seta medially, with 2 robust setae on inferodistal corner; carpus inferior margin with 4 denticles, 3 robust setae and 1 submarginal simple seta, superior margin with 1 simple seta on distal corner; propodus longer than carpus, with 3 robust setae on inferior margin; dactylus with 1 subapical simple seta, unguis subequal in length to dactylus, accessory claw length 0.6 unguis length.

***Pereopod 2*** (Fig. 2B) basis about 3.5 times as long as greatest width; ischium about 0.9 times as long as basis, with 1 long seta on inferior margin; merus about 0.5 times as long as carpus, inferior margin with 2 and superior margin with 1 long simple setae; carpus and propodus subequal in length, carpus inferior margin with 3 robust setae, superior margin with 1 seta medially and 2 simple long setae on distal corner; propodus inferior margin with 3 robust setae and 1 long simple seta, superior margin with 3 long simple setae; dactylus with 2 subapical simple setae, a small accessory claw, unguis 1.5 times as long as dactylus.

***Pereopod 5*** (Fig. 2C) basis about 3.8 times as long as greatest width; ischium about 0.92 times as long as basis, with 1 long seta on inferior margin and 2 long setae on superior margin; merus about 0.46 times as long as carpus, inferior and superior margin with 2 simple setae each; carpus shorter than propodus, with 2 robust setae on inferior margin and 1 simple seta on superior margin medially; propodus inferior margin with 3 robust setae, superior margin with 3 simple setae; dactylus with 2 subapical simple setae; unguis 1.4 times as long as dactylus.

***Pereopod 7*** (Fig. 2D) basis about 3.8 times as long as greatest width; ischium about 0.85 times as long as basis, with 1 long seta on inferior margin; merus about 0.47 times as long as carpus, inferior and superior margins with 1 long simple seta; carpus shorter than propodus, with 2 robust setae on inferior margin; propodus inferior margin with 2 robust setae, superior margin with 3 simple setae; unguis 1.5 times as long as dactylus.

***Pleopod 1*** (Fig. 3A) distally separate, with 2–4 apical setae; short lateral projections present, each projection with 1–2 setae.

***Pleopod 2*** (Fig. 3B) protopod length 2.4 width, with 16 simple marginal setae; second article of endopod elongate, downwardly curved, proximally swollen, becoming narrower until distal pointed apex, about 2.4 as long as protopod, without any ornamentation.

***Pleopod 3*** (Fig. 3C), endopod with 2 long plumose setae apically and 1 long plumose seta laterally; exopod composed of two articles, narrower than endopod; article 2 with 1 terminal seta.

***Penes*** (Fig. 3D) fused along basal part, well separated at distal part, tapering to narrowly rounded apices.

**Female** (Figs 1A, 4B, 5A, B). Similar to males in the morphology of all pereonal appendages. Body broader than in males, about 1.8 times as long as wide; widest at pereonite 3. The largest female length is 1.34 mm. ***Operculum*** (Fig. 3E) ovate, 1.2 times as long as broad, without any marginal setae.

##### Remarks.

*Heterosignumbehbehanii* sp. nov. is most similar to *H.elegans* Shimomura & Mawatari, 2002 from Japan, as both of these species have lateral spine-like processes on pereonites 2–7, a pleotelson with 6 denticles on its lateral margins of the posterior part, and pleopod 2 with a slender very long, curved second article of the endopod. However, the new species is distinguished from *H.elegans* in having its notably shorter lateral spine-like processes on pereonites 2–7, a smaller anterior part of the pleotelson (about 0.1 vs. 0.4 times the total length of pleotelson), and the first pleopod being distally separate instead of distally connected.

The new species differs from *Heterosignumunicornis* (Kensley, 1976), the only species reported from the Indian Ocean (southern Indian Ocean, Amsterdam Island). *Heterosignumunicornis* has a stout mid-dorsal spine (horn) on the first pereonite, lacking lateral spine-like processes on pereonites 5–7, and a pleotelson with 4 denticles on its lateral margins.

### ﻿Key to species of *Heterosignum* (males)

**Table:** 

1	Pereonite 1 with strong mid-dorsal horn-shape spine	**2**
–	Pereonite 1 without mid-dorsal horn-shape spine	***3***
2	Head with one stout mid-dorsal horn-shape spine; pereonite 1 with lateral spine-like processes	***H.ohtsukai* Shimomura & Mawatari, 2002**
–	Head without mid-dorsal horn-shape spine; pereonites 1 and 7 without lateral spine-like processes	***H.unicornis* (Kensley, 1976)**
3	Head without long anteriorly directed processes on the anterior margin	**4**
–	Head with 2 long anteriorly directed processes on the anterior margin	***H.bicornis* Shimomura, 2011**
4	Eyestalks slender, medium or long, with a few ommatidia	**5**
–	Eyestalks stout, short, without ommatidia	***H.hashimotoi* Shimomura, 2009**
5	Pereonites 2–4 and 6 with a pair of relatively short single lateral spine-like processes or not; anterior part of the pleotelson short, cylindrical, about 0.1 of the total length	**6**
–	Pereonites 2–4 and 6 with a pair of very long single lateral spine-like processes; anterior part of the pleotelson long, cylindrical, about 0.4 of the total length	***H.elegans* Shimomura & Mawatari, 2002**
6	Pereonites 4, 5 and 7 without lateral spine-like processes; pleopod 2 with broad protopod, bearing 12 marginal setae, endopodal second article about 1.1 as long as protopod	***H.mutsuensis* Gamô, 1976**
–	Pereonites 2–7 with short lateral spine-like processes; pleopod 2 with broad protopod, bearing 16 marginal setae, endopodal second article about 2.4 as long as protopod	***H.behbehanii* sp. nov.**

#### ﻿Family Munnidae Sars, 1897

##### ﻿Genus Uromunna

Taxon classificationAnimaliaIsopodaMunnidae

Menzies, 1962

B52182D9-B853-5BDF-B838-34F79E366411

###### Type species.

*Uromunnaubiquita* (Menzies, 1952), by original designation.

###### Diagnosis.

The most recent diagnosis to the genus is that of Esquete and Wilson (2016).

##### ﻿ Uromunna alyamanii sp. nov.

Taxon classificationAnimaliaIsopodaMunnidae

BDCDBFA1-FF35-530D-98D9-D3F369154BE1

https://zoobank.org/383D6E48-C576-431E-92C8-29C99BE55CBD

Figs 5C , 6 , 7 , 8

###### Etymology.

The species is named in honour of Dr Faiza Yousef Al-Yamani, a pioneering figure who established the Oceanography Program at the Environment and Life Sciences Research Center at Kuwait Institute for Scientific Research (KISR) in 1991.

###### Type material.

***Holotype***: • ♂, 0.78 mm (ZMH-K-64937); KUWAIT. Quaruh Island; 28°49.105'N, 48°46.553'E; 10. XI. 2014; V. Grintsov leg. ***Paratypes***: • 1♀, 0.88 mm (ZMH-K-64938), same data as the holotype • 1♀, 0.78 mm (ZMH-K-64939), Al-Khiran; 29°38'48.47"N, 48°23'28.68"E; 05. I.2015; V. Grintsov leg. • 2 ♀♀, up to 0.75 mm (ZMH-K-64940), Auha Island; 29°22.32'N, 48°26.27'E; 10. II.2016; V. Grintsov leg. • 2 ♀♀, up to 0.8 mm, 1♂, 0.725 mm, 1 slide (ZMH-K-64941), Al-Nuwaiseeb; 28°34.792'N, 48°24.078'E; 07.I.2015; V. Grintsov leg. • 1♀, 0.787 mm, 1 slide (ZMH-K-64941), Failaka Island; 29°28.049'N, 48°17.838'E; 22.XII.2014; V. Grintsov leg.

###### Diagnosis.

Pleotelson 1.26 times longer than wide, 0.31 times of whole body, and lateral margins each with 2 denticles. Maxillipedal endite with three coupling hooks. Male pleopod 1 distal margin medial lobes convex, each lobe with three setae. Pleopod 2 protopod elongate, with distally rounded apex; endopod stylet extending just beyond apex of protopod, about 0.7 times as long as protopod. Pleopod 4 exopod distal tip with 1 elongate plumose seta.

###### Description.

**Figure 6.**
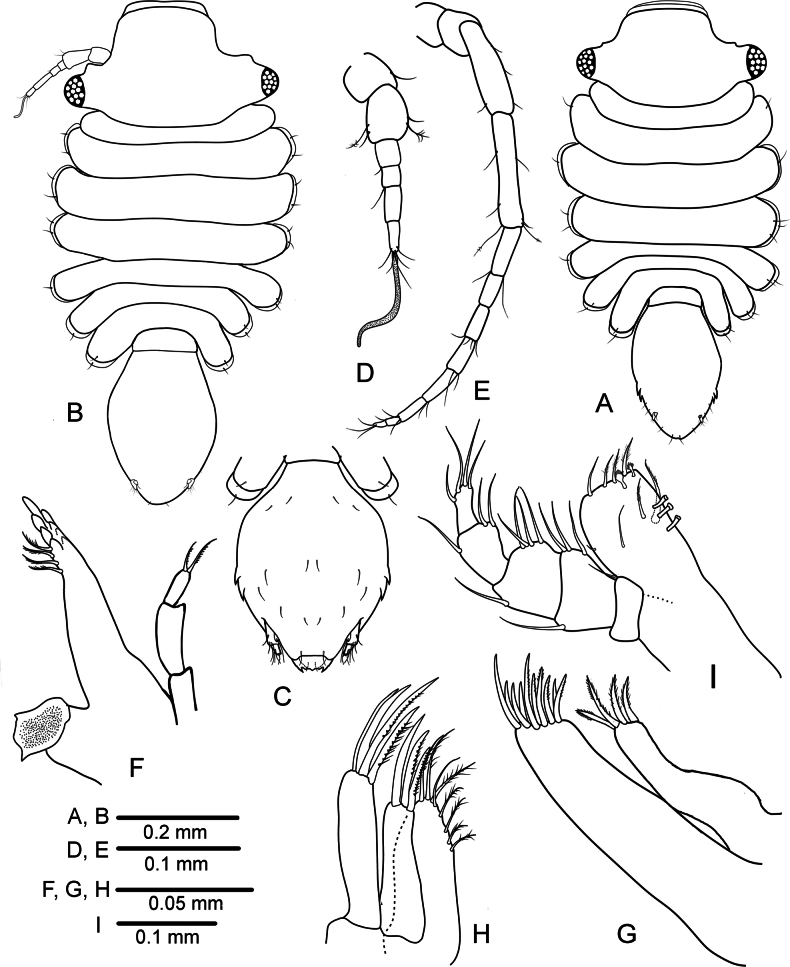
*Uromunnaalyamanii* sp. nov. **A** female habitus **B–I** male holotype (ZMH-K-64937) **B** habitus **C** pleotelson **D** antennula **E** antenna **F** mandible **G** maxillula **H** maxilla **I** maxilliped.

**Male: *Body*** (Fig. 6B) length 0.78 mm; about 2.1 times as long as wide.

***Head*** (Fig. 6B) 0.65 times as long as wide; anterior margin straight; preocular lobes distinct and projecting. ***Eyes*** present, ommatidia on eye lobe outer margin, with 12 ommatidia.

***Pereon*** (Fig. 6B) dorsal surface smooth; ***pereonite 1*** narrowest; ***pereonite 3*** widest and longest; coxae visible on pereonites 2–7, each with 1–2 small setae; ***pereonites 5*–*7*** turned back; lateral margins rounded; ***pereonite 7*** longer then pereonite 6, about 1.5 times as long as pereonite 6.

***Pleonite 1*** small, about 0.4 times as long as wide, without dorsal setae.

***Pleotelson*** (Figs 6B, 5C, 6C) about 1.26 times longer than wide, pyriform, lateral margins each with 2 small lateral denticles; dorsal surface covered with 16 simple setae.

***Uropod*** (Fig. 6C) cylindrical, endopod bearing 6 lateral and apical setae; exopod minute, with one simple seta.

***Antennula*** (Fig. 6D) peduncular article 1 shorter than article 2; article 2 with 2 simple and 2 small sensory palmate setae sub distally; article 3 about 0.5 length of article 2, with 1 simple seta distally; flagellum with three articles, article 2 longest; article 3 distally with 1 long aesthetasc.

***Antenna*** (Fig. 6E) about 0.8 times as long as body; antennal flagellum shorter than peduncular articles, with 8 articles.

***Mandible*** (Fig. 6F) incisor process and ***lacinia mobilis*** with 4 cusps; spine row with 4 robust serrate setae. Palp articles 1 and 2 without setae, third article 0.55 times as long as article 2, with 2 serrated setae apically.

***Maxillula*** (Fig. 6G) lateral lobe with 10 robust apical setae; medial lobe with 4 robust serrate setae on distal margin.

***Maxilla*** (Fig. 6H) lateral and mesial lobes each with 4 and 3 pectinate robust apical setae; medial lobe with 8 robust setae along distal margin, some of them serrated.

***Maxilliped*** (Fig. 6I) endite distal margin with 2 fan-shaped setae, 4 serrate setae, and 3 sub marginal serrated setae dorsally; mesial margin with 3 coupling hooks; palp article 1 bearing short seta on distomesial corner; article 2 sub equal length to article 3, with 3 setae medially and 1 seta on distolateral corner; article 3 with 4 setae medially and 1 seta laterally; article 4 subequal in length to article 3, with 2 setae distomedially and 1 seta laterally; article 5 with 4 setae distally.

**Figure 7.**
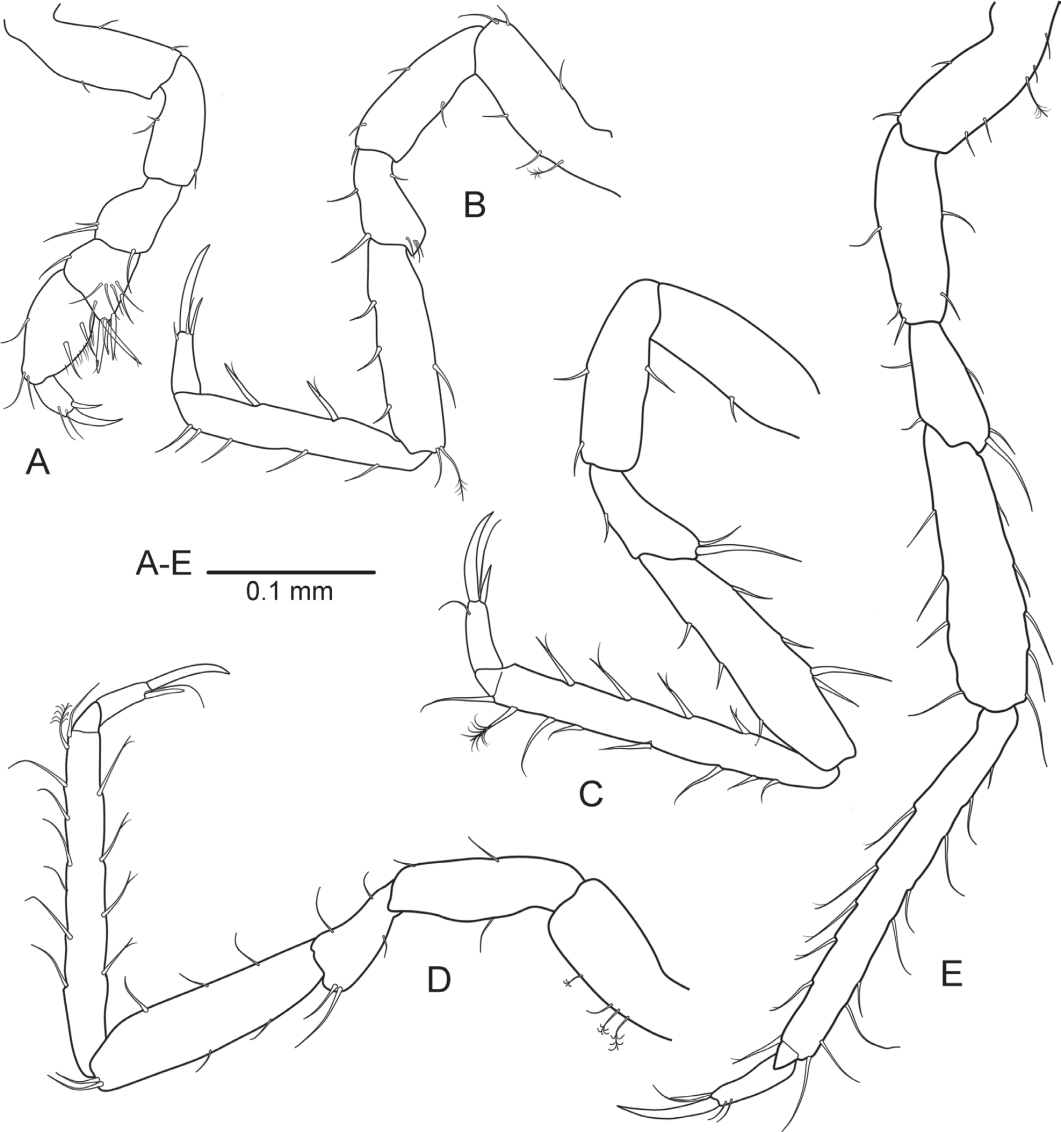
*Uromunnaalyamanii* sp. nov. male holotype (ZMH-K-64937) **A** pereopod 1 **B** pereopod 2 **C** pereopod 4 **D** pereopod 5 **E** pereopod 7.

***Pereopod 1*** (Fig. 7A) shorter than ***pereopods 2*–*7***; basis 2.6 times as long as greatest width, superior margin with 1 and inferior margin with 2 small setae; ischium about 0.7 times as long as basis, superior margin with 2 and inferior margin with 1 small setae; merus longer than carpus, 0.75 times as long as ischium, superior margin with 2 and inferior margin with 1 robust setae distally; carpus with 4 robust bifid setae on inferodistal corner, inferior margin with 1 marginal and 4 submarginal long simple setae, superior margin with 1 long simple seta medially; propodus 1.75 times as long as carpus, inferior margin fringed with fine setae, bearing 2 robust submarginal setae, superior margin with 1 long simple seta medially and 2 simple setae distally; dactylus with 2 subapical simple setae, a long unguis, and a long accessory claw.

***Pereopod 2*** (Fig. 7B) basis about 3.1 times as long as greatest width; ischium about 0.7 times as long as basis, with 3 long setae on inferior margin and 1 long seta on superior margin; merus about 0.45 times as long as carpus, inferior margin with 2 and superior margin with 4 simple setae; carpus about 0.9 times as long as propodus, inferior margin with 3 robust setae, superior margin with 1 seta medially and 1 simple and 1 plumose setae distally; propodus inferior margin with 2 robust bifid setae, superior margin with 5 long simple setae; dactylus with a narrow accessory claw, unguis longer than dactylus.

***Pereopod 4*** (Fig. 7C) similar to ***pereopod 5*** (Fig. 7D), propodus about 1.5 times as long as carpus, with 4 robust bifid setae on inferior margin and 7 long simple setae and 1 sensory palmate seta on superior margin.

***Pereopod 7*** (Fig. 7E) basis about 2.8 times as long as greatest width; ischium about 0.86 times as long as basis, with 3 long setae on inferior margin and 2 long setae on superior margin; merus about 0.46 times as long as carpus; carpus with 4 robust setae on inferior margin and 5 robust setae on superior margin; propodus about 1.5 times as long as carpus, inferior margin with 6 robust bifid setae, superior margin with 8 long simple setae and 1 sensory palmate seta; unguis 1.24 times as long as dactylus.

**Figure 8.**
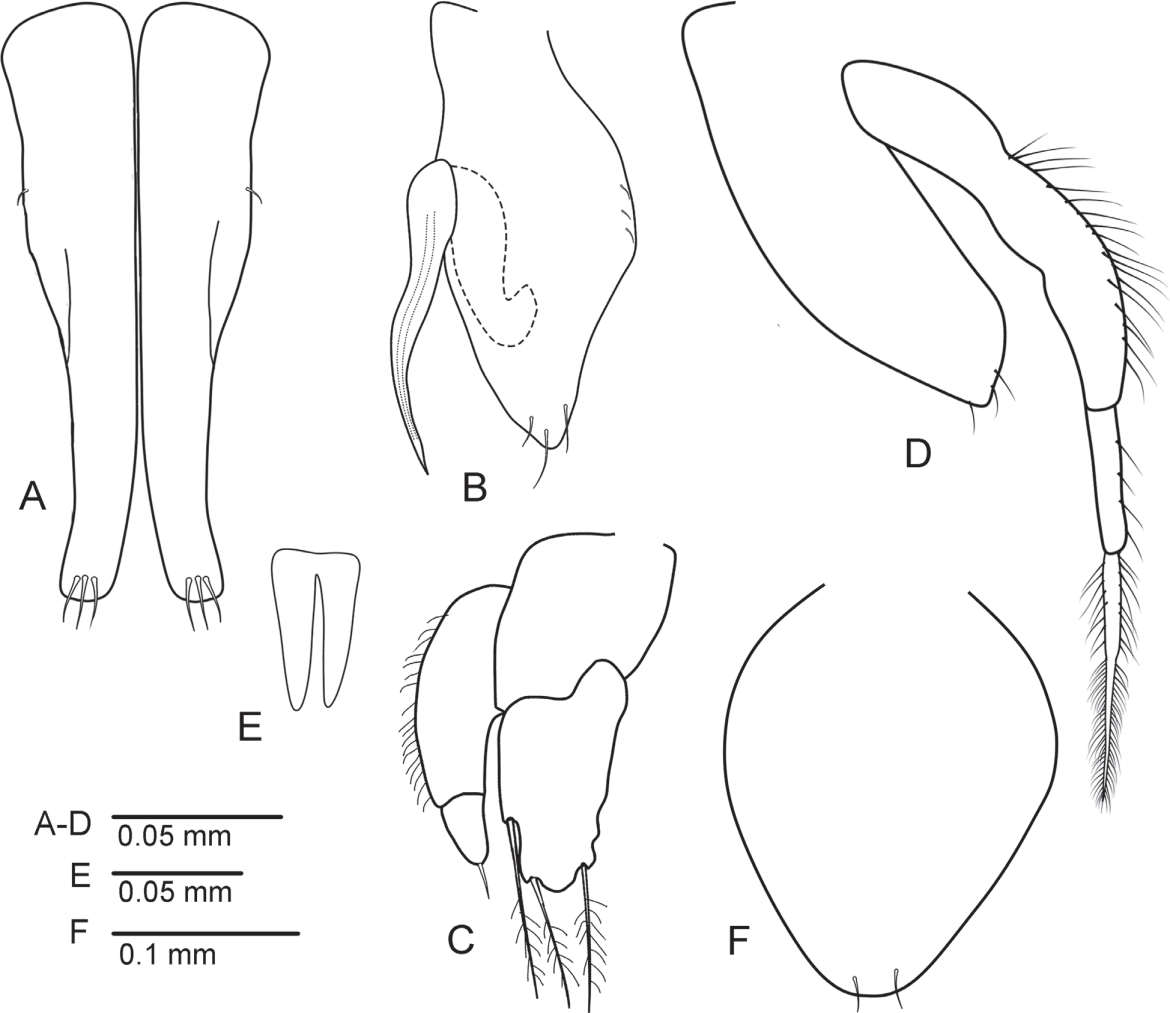
*Uromunnaalyamanii* sp. nov. male holotype (ZMH-K-64937) **A** pleopods 1 **B** pleopod 2 **C** pleopod 3 **D** pleopod 4 **E** penial processes **F** female operculum.

***Pleopod 1*** (Fig. 8A) consists of two coalescent halves, each half 4.5 times longer than maximum width; proximal part enlarged and tapering distally, distal margin narrowly rounded, each lobe with three setae distally.

***Pleopod 2*** (Fig. 8B) protopod elongate, with distally rounded apex, about 2.2 times longer than wide, with 3–4 subapical setae; endopodal stylet, elongate, medially swollen but becoming narrower until distal tip, without ornamentation, extending just beyond apex of protopod, endopod stylet length about 0.7 protopod lengths.

***Pleopod 3*** (Fig. 8C) protopod as long as width; endopod 1.8 times longer than wide, with three plumose setae apically; exopod with two articles, proximal article 2.4 longer than wide, lateral margin fringed with fine setae, distal article 1.6 times longer than wide, with 1 simple seta distally.

***Pleopod 4*** (Fig. 8D) exopod with 2 articles, article 1 lateral margin fringed with dense fine setae, article 2 lateral margin with a few fine setae; with 1 elongate plumose seta distally.

***Penes*** (Fig. 8E) fused along basal part, well separated at distal part, tapering to rounded apices.

**Female** (Figs 5C, 6A) body about 1.9 times longer than wide; widest at pereonite 3. Largest female length 0.74 mm. Female operculum (Fig. 8A) length 1.25 width, with one pair of subterminal elongate setae.

###### Remarks.

*Uromunnaalyamanii* sp. nov. shares similarities with *U.naherba* Esquete, Wilson & Troncoso, 2014 from NW Iberian Peninsula, Spain, and *U.jejuensis* Kim, Lee & Karanovic, 2023 from the Mun Island, Sea of Japan. These similarities include the appearance of mouthparts, pereopods, and pleopods, especially a round distal apex of male pleopod 1 with three pairs of apical setae, the presence of denticles on the pleotelsonic lateral margins, and a female operculum with a terminal pair of setae. However, the present species also have distinct differences, such as a pleotelson with 2 lateral margin denticles on each side, a feature not found in the latter species.

The new species differs from *U.sheltoni* (Kensley, 1977) from South Africa, which has the distally concave pleopod 1, with 4 short apical setae (vs. distally rounded, with 3 short setae in the present species), and the shorter endopodal stylet of pleopod 2, which does not reach beyond the apex of the ramus.

### ﻿Key to species of the Indian Ocean species of *Uromunna* (males)

**Table:** 

1	Pleotelson lateral margins denticles absent	**2**
–	Pleotelson lateral margins denticles present, 2 denticles on each side	***U.alyamanii* sp. nov.**
2	Pleopod 1 strongly curving laterally at apices, each apex with 2 pairs of setae along free mesial margin	***U.brevicornis* (Thomson, 1946)**
–	Pleopod 1 evenly tapering to apices; each apex with 3 subterminal setae	**3**
3	Pleopod 1 with rounded-truncate apices; pleopod 2 with acute apex, bearing 2 setae along mesial edge	***U.humei* Poore, 1984**
–	Pleopod 1 with obliquely truncate apices; pleopod 2 with rounded-acute apex, bearing 1 subterminal seta	***U.phillipi* Poore, 1984**

## Supplementary Material

XML Treatment for Heterosignum

XML Treatment for Heterosignum behbehanii

XML Treatment for Uromunna

XML Treatment for Uromunna alyamanii

## ﻿Additional information

### Author ORCIDs

Valiallah Khalaji-Pirbalouty https://orcid.org/0000-0002-0892-7463

Manal Abdulrahman Al-Kandari https://orcid.org/0000-0003-0073-7929

### Data availability

All of the data that support the findings of this study are available in the main text.
